# INO80E Suppresses Oxidized LDL-Induced Endothelial Apoptosis Through HDAC1-Mediated Stabilization of YY1

**DOI:** 10.3390/biom16081174

**Published:** 2026-08-12

**Authors:** Tingting Liu, Quanye Luo, Shihong Yang, Dongmei Yang, Xuzhen Lv, Liyan Zhao, Qinhui Tuo

**Affiliations:** 1 Key Laboratory for Quality Evaluation of Bulk Herbs of Hunan Province, School of Pharmacy, Hunan University of Chinese Medicine, Changsha 410208, China; 2 School of Pharmacy, Hunan University of Medicine, Huaihua 418000, China; 3 Basic Research Center of Integrated Chinese and Western Medicine on Prevention and Treatment of Vascular Diseases, Medical School, Hunan University of Chinese Medicine, Changsha 410208, China

**Keywords:** atherosclerosis, endothelial cells, apoptosis, INO80E, HDAC1, YY1, deacetylation

## Abstract

Objectives: Endothelial apoptosis is a central event in atherosclerotic vascular injury, yet the contribution of the INO80 chromatin-remodeling subunit INO80E to this process remains unclear. This study examined whether INO80E is altered during atherosclerosis (AS)-associated endothelial injury and explored its functional role in ox-LDL-triggered apoptosis. Methods: Atherosclerotic mouse models were used to examine INO80E expression in vascular tissues, and ox-LDL-treated HUVECs were applied as an in vitro model of endothelial injury. The functional role of INO80E was evaluated using lentiviral overexpression and siRNA-mediated knockdown approaches, while apoptosis was measured by flow cytometry and TUNEL staining. Mechanistic experiments included co-immunoprecipitation, immunofluorescence, YY1 acetylation analysis, and pharmacological inhibition with the pan-HDAC inhibitor trichostatin A (TSA). In addition, YY1-silencing rescue experiments were performed in INO80E-overexpressing cells to determine whether YY1 contributes to the protective effect of INO80E against ox-LDL-induced endothelial apoptosis. Results: INO80E expression was reduced in the endothelial layer of ApoE^−/−^ aortas and in ox-LDL-treated HUVECs. INO80E overexpression attenuated ox-LDL-induced apoptosis and increased the Bcl-2/BAX ratio, whereas INO80E knockdown produced the opposite effect. INO80E colocalized with HDAC1, increased the HDAC1-YY1 association, decreased YY1 acetylation, and prolonged YY1 protein stability. TSA treatment weakened the anti-apoptotic phenotype associated with INO80E overexpression, and rescue experiments showed that YY1 knockdown partially reversed the INO80E overexpression-associated regulation of Bcl-2 and BAX. Conclusions: These findings suggest that INO80E protects endothelial cells from ox-LDL-induced apoptosis, at least in part by promoting HDAC-associated YY1 deacetylation and stabilization. The INO80E-HDAC1-YY1 pathway may represent a candidate protective mechanism in AS that requires further validation.

## 1. Introduction

Atherosclerosis (AS), a chronic inflammatory disease of the arteries, remains the leading cause of global cardiovascular mortality and morbidity, despite the widespread use of lipid-lowering therapies and surgical interventions [1,2]. This pathology is initiated by vascular endothelial dysfunction and apoptosis, which compromise intimal integrity and facilitate inflammatory cell infiltration [3,4]. Among the various pathogenic stimuli, oxidized low-density lipoprotein (ox-LDL) promotes endothelial injury by inducing oxidative stress and apoptotic signaling, thereby contributing to endothelial cell death and plaque progression [5,6]. In this process, the Bcl-2/BAX ratio is commonly used to reflect apoptotic susceptibility in endothelial cells [7]. Because endothelial injury occurs early in AS and may be modifiable, defining regulators that preserve endothelial viability under ox-LDL stress remains biologically important [8].

Epigenetic regulation is a key component of vascular homeostasis and disease [9]. Among epigenetic mechanisms, ATP-dependent chromatin remodeling complexes regulate gene accessibility by altering nucleosome structure and have been implicated in cardiovascular disease [10]. The INO80 chromatin remodeling complex is a multi-subunit ATP-dependent remodeler with established roles in DNA repair, replication, and transcriptional regulation [11]. We previously identified INO80E, a core component of this complex, as a pleiotropic risk gene associated with schizophrenia-related phenotypes [12]. Whether INO80E also contributes to vascular endothelial injury has not been determined.

INO80-related proteins have been reported to functionally associate with YY1 (Yin Yang 1) [13,14], but the relevance of this relationship in endothelial cells is unknown. YY1 is a transcription factor involved in cell survival, including regulation of anti-apoptotic genes such as Bcl-2, and endothelial YY1 loss has been linked to vascular abnormalities [15]. YY1 protein stability is influenced by acetylation: acetylated YY1 is more susceptible to ubiquitin-dependent proteasomal degradation, whereas deacetylation favors stabilization [16]. HDAC1, a deacetylase that regulates histones and non-histone substrates, has also been connected to endothelial stress responses [17,18]. These observations led us to test whether INO80E affects endothelial survival in association with HDAC1-dependent control of YY1 acetylation and stability.

Accordingly, we examined INO80E expression in atherosclerotic aortic endothelium and ox-LDL-injured HUVECs and used gain-of-function, loss-of-function, and rescue approaches to determine whether INO80E regulates endothelial apoptosis and the Bcl-2/BAX balance through an HDAC1/YY1-dependent pathway.

## 2. Materials and Methods

### 2.1. Animal Tissue Samples

Aortic specimens used in this study were retrieved from an established mouse model of atherosclerosis. Male ApoE^−/−^ mice (*n* = 40; 6–8 weeks of age; 22.9–24.8 g) and age-matched male SPF C57BL/6 mice (*n* = 10; 21.8–23.5 g) were supplied by the Laboratory Animal Center of Hunan University of Chinese Medicine, Changsha, China. Following a 7-day adaptation period, all animals were housed under controlled conditions, including a temperature of 25 ± 1 °C, relative humidity of 60% ± 10%, and a 12 h light/12 h dark schedule, with unrestricted access to food and drinking water. C57BL/6 mice were maintained on a normal chow diet, whereas ApoE^−/−^ mice received a high-fat diet for 12 weeks, composed of 77.5% basal chow, 20% lard, 2% cholesterol, and 0.5% sodium cholate. All animal procedures complied with the ARRIVE 2.0 guidelines and the Guide for the Care and Use of Laboratory Animals, and were authorized by the Ethics Committee of Hunan University of Chinese Medicine (approval number LL2023033101).

After completion of the dietary intervention, mice were deprived of food for 12 h but allowed free access to water. They were then anesthetized by intraperitoneal injection of pentobarbital sodium at 50 mg/kg and euthanized during vascular perfusion. The right atrium was opened, ice-cold saline was infused through the left ventricle until organ blanching was evident, and the aortas were harvested and fixed in 4% paraformaldehyde. Plaques in this cohort had been confirmed previously by Oil Red O and histology [19]. For the present staining, control and high-fat diet ApoE^−/−^ aortic samples (*n* = 5/group) were selected only when morphology, perfusion, fixation, and, for ApoE^−/−^ tissues, lesion confirmation was satisfactory.

### 2.2. Cell Culture and Treatments

Primary HUVECs (No. CTCC-0804-PC; donor data not provided) were purchased from Meisen Cell Technology Co., Ltd. (Jinhua, China). Cells were maintained at 37 °C using ECM medium (Meisen, Jinhua, China) supplemented with 5% fetal bovine serum, 1% endothelial cell growth supplement, and 1% penicillin–streptomycin. Experiments used passages 3–5.

Highly oxidized human LDL came from Yiyuan Biotechnologies (Guangzhou, China; No. YB-002-1). The supplier reported CuSO_4_ oxidation, 2.5-fold relative electrophoretic mobility versus native LDL, and TBARS of 90.0 ± 9.9 nmol MDA/mg protein. The PBS/0.2 mM EDTA-Na2 stock was stored at 4 °C and used within 3 weeks without freezing. Trichostatin A (TSA) [20], a pan-HDAC inhibitor (Proteintech, Wuhan, China; No. CM00558) was dissolved in DMSO.

Experimental grouping was arranged as follows:
**Category****Groups****Treatment Conditions**ControlsUntreated control; vehicle controlComplete medium only, or the corresponding ox-LDL diluent or DMSO. DMSO was kept below 0.1%.Pharmacological treatmentox-LDL; TSA; ox-LDL + TSAox-LDL, 50 μg/mL for 24 h; TSA, 2.5 μM for 24 h, alone or with ox-LDL.INO80E overexpressionox-LDL + Vector; ox-LDL + oe-INO80EVector- or oe-INO80E-transduced HUVECs were treated with ox-LDL for 24 h.INO80E knockdownsi-NC; si-INO80E; si-NC + ox-LDL; si-INO80E + ox-LDLsi-NC or si-INO80E was introduced under basal conditions or followed by 24 h ox-LDL exposure.YY1 rescueVector + si-NC + ox-LDL; Vector + si-YY1 + ox-LDL; oe-INO80E + si-NC + ox-LDL; oe-INO80E + si-YY1 + ox-LDLVector or oe-INO80E transduction was combined with si-NC or si-YY1 followed by 24 h ox-LDL exposure.

### 2.3. Lentiviral Transduction for INO80E Overexpression

INO80E-overexpressing and empty-vector lentiviruses were obtained from HySigen Biosciences (Suzhou, China). Log-phase HUVECs at 60–70% confluence were infected in fresh endothelial basal medium at MOI 50 with polybrene (5 μg/mL). After 24–48 h, the virus-containing medium was replaced with complete medium. INO80E upregulation was confirmed by qRT-PCR and Western blotting before downstream assays.

### 2.4. siRNA Transfection

INO80E or YY1 knockdown was performed with Sangon Biotech (Shanghai, China) siRNA transfection reagent. For rescue assays, si-YY1 was combined with vector or oe-INO80E lentiviral transduction before ox-LDL exposure. All siRNAs (Sangon Biotech, Shanghai, China) were used at 20 nM: si-INO80E sense, 5′-GCGCAAAGGAAAUUACUGAdTdT-3′; si-INO80E antisense, 5′-UCAGUAAUUUCCUUUGCGCdTdT-3′; si-YY1 sense, 5′-CCUGAUUAUUCAGAAUAUAdTdT-3′; si-YY1 antisense, 5′-UAUAUUCUGAAUAAUCAGGdTdT-3′.

### 2.5. Quantitative Real-Time PCR

Total RNA was extracted with Accurate Biology reagent (Changsha, China; No. AG21023), checked by A260/A280, and reverse-transcribed using a Novoprotein kit (Suzhou, China; No. 0535004). qPCR was run on a Roche LightCycler^®^ 96 (Roche Diagnostics, Basel, Switzerland) with SYBR Green qPCR Master Mix (Novoprotein; No. 05384005). Each reaction contained 2× mix, primers (0.2–1.0 μM), cDNA, and RNase-free water. Cycling was 95 °C (30 s), then 30 cycles of 95 °C (20 s), 50–60 °C (20 s), and 72 °C (30 s), followed by melting analysis. Expression was calculated by 2^−ΔΔCt^ using GAPDH. QPCR assays were performed with independent biological replicates, and technical wells were averaged before statistical analysis. Primer sequences were: INO80E forward, CTGCCCCGGAAACTCAAGAT; INO80E reverse, CTGCATCGCTGAACATCTGC; YY1 forward, ACGGCTTCGAGGATCAGATTC; YY1 reverse, TGACCAGCGTTTGTTCAATGT; GAPDH forward, AGTGATGGCATGGACTGTGG; GAPDH reverse, GATTTGGTCGTATTGGGCGC.

### 2.6. Western Blotting and Co-Immunoprecipitation

Western Blotting: Proteins from cells were prepared in RIPA buffer and quantified by BCA. Samples (15–20 μg) were separated on 12–15% SDS-PAGE and transferred 100 V (90 min). Membranes were blocked in TBST containing 5% non-fat milk for 1 h and then incubated with the indicated primary antibodies overnight at 4 °C, as follows: INO80E (Santa Cruz, Shanghai, China;sc-515298), HDAC1 (Proteintech, Wuhan, China; 10197-1-AP), YY1 (Proteintech, 22156-1-AP), acetyl-lysine (PTM Biolabs, Hangzhou, China; PTM-101), Bcl-2 (Proteintech, 12789-1-AP; ZEN-BIOSCIENCE, Chengdu, China; R23309), BAX (Proteintech, 50599-2-Ig), GAPDH (Proteintech, 60004-1-Ig), or Tubulin (Proteintech, 80762-1-RR), each at 1:1000, and then exposed to HRP-conjugated secondary antibodies (Proteintech, SA00001-1/2; 1:10,000). ECL signals were analyzed in ImageJ (version 2.16.0) with Tubulin, β-Actin, or GAPDH as loading controls.

Co-Immunoprecipitation (Co-IP): For each reaction, 500 μg of clarified lysate was incubated with 10 μg target antibody or matched IgG at 4 °C overnight; 10% lysate was kept as input. Protein A/G magnetic beads (30 μL) (ACE Biotechnology, Nanjing; No. BK0004-02) were subsequently added and incubated overnight. The beads were washed six times, and the bound proteins were then eluted for Western blot analysis.

### 2.7. Cell Viability Assay

HUVECs were seeded in 96-well plates (8000 cells/well, 100 μL), treated as indicated, incubated with 10 μL CCK-8 for 1 h at 37 °C in the dark, and read at 450 nm. Background-corrected values were normalized to controls; each biological experiment contained six technical wells.

### 2.8. Apoptosis Assays

Flow Cytometry: Apoptosis was quantified using an Annexin V-FITC/PI kit (4A Biotech, Suzhou, China; No. FXP018). Cells were collected, PBS-washed, resuspended in binding buffer, stained for 15 min in the dark, and analyzed on a CytoFLEX S cytometer (Beckman Coulter, Brea, CA, USA). At least 10,000 events were acquired, with debris excluded and compensation set using unstained and single-stained controls.

TUNEL Staining: Apoptotic nuclei were detected with the Beyotime TUNEL kit (Shanghai, China; No. C1090). Cells were fixed with 4% paraformaldehyde for 15 min, permeabilized with 0.3% Triton X-100 for 5 min, reacted with TUNEL mixture, counterstained with DAPI, and imaged on an Olympus BX53 microscope (Olympus, Tokyo, Japan). Three random fields per sample were counted by a blinded investigator.

### 2.9. Immunofluorescence

For tissue immunofluorescence, paraformaldehyde-fixed aortic arches were OCT-embedded and cut at 5 μm. Three to five non-consecutive sections per mouse were blocked, incubated at 4 °C with anti-INO80E and anti-CD31 (Proteintech, Wuhan, 11265-1-AP; 1:200), labeled with Alexa Fluor-conjugated secondary antibodies (1:500), counterstained with DAPI, and imaged under uniform exposure settings. INO80E-positive nuclei within CD31-positive endothelium were counted in ImageJ (version 2.16.0) from 3–5 random high-power fields across 3–5 non-consecutive sections per mouse and normalized to endothelial area; analysis was blinded.

For cultured-cell immunofluorescence, coverslip-grown HUVECs were fixed, permeabilized, blocked with 5% BSA, incubated with anti-INO80E and anti-HDAC1 (both 1:200) at 4 °C overnight, labeled with Alexa Fluor-conjugated secondary antibodies (1:500), stained with DAPI, and imaged using a Nikon A1 confocal microscope (Nikon Corporation, Tokyo, Japan).

### 2.10. Protein Stability Assay

YY1 protein stability was examined by treating HUVECs with CHX (0.1 μg/mL), collecting cells at scheduled times, and measuring YY1 by Western blotting.

### 2.11. Statistical Analysis

Data are mean ± SD. Unless stated otherwise, *n* indicates independent biological replicates. For tissue immunofluorescence, each mouse was one replicate; for cell experiments, independent cultures on different days were replicates, with technical wells or image fields averaged before analysis. Two groups were compared with two-tailed Student’s *t*-test, and multiple groups with one-way ANOVA plus Tukey’s test. *p* < 0.05 was considered significant. GraphPad Prism (version 10.3.0) was used.

### 2.12. Use of Generative AI Tools

During manuscript preparation, DeepSeek (DeepSeek-V3, accessed in July 2026) was used to assist with English language editing, grammar correction, wording refinement, and text organization.

## 3. Results

### 3.1. INO80E Expression Is Reduced in the Aortic Endothelium of Atherosclerotic Mice

**Figure 1:**
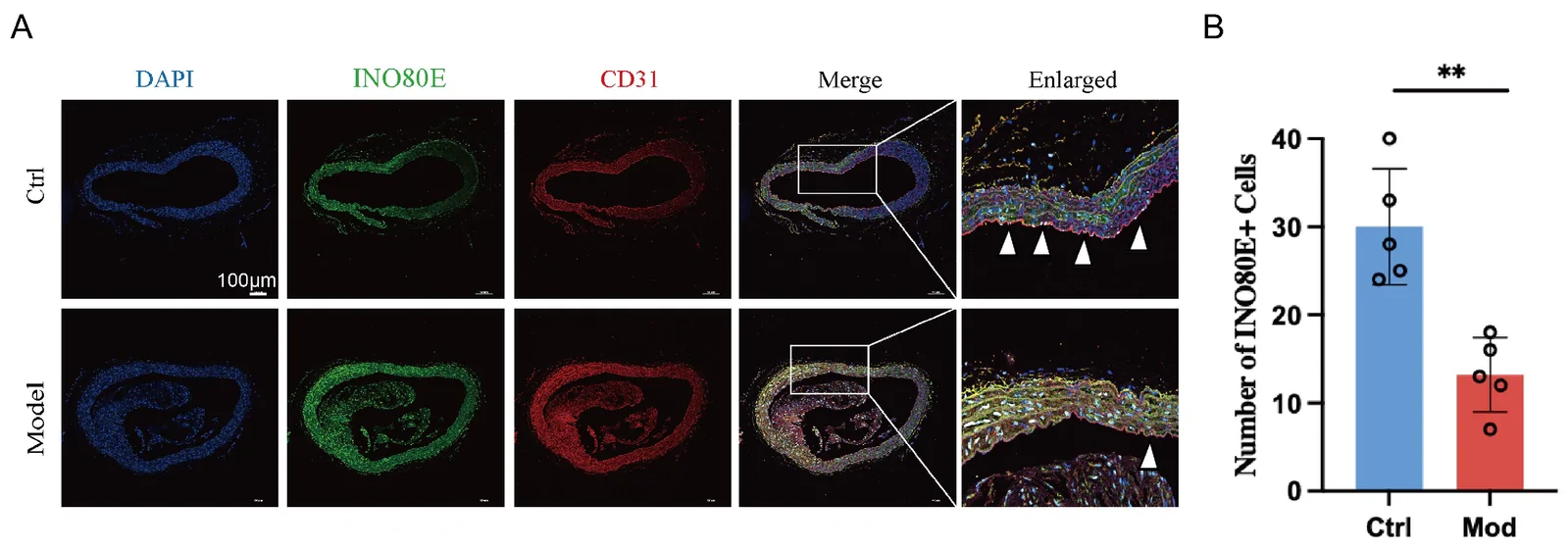
Endothelial INO80E signal in control and atherosclerotic aortas. (**A**) Aortic arch sections stained for INO80E (green), CD31 (red), and DAPI (blue); boxed regions are enlarged, and arrowheads mark endothelial INO80E. Scale bar, 100 μm. (**B**) Quantified INO80E-positive cells in the CD31-positive layer. *n* = 5. ** *p* = 0.0014.

To explore the role of INO80E in AS, we first examined its expression in the aortic endothelium of ApoE^−/−^ mice fed a high-fat diet. INO80E colocalized with CD31 in the aortic endothelium of control mice, whereas its fluorescence in the CD31-positive endothelial layer was markedly reduced in ApoE^−/−^ mice (Figure 1A, white arrowheads). Quantitative analysis confirmed a significant reduction in the number of INO80E-positive endothelial cells in the atherosclerotic group (Figure 1B), suggesting that endothelial INO80E expression may be reduced during AS.

### 3.2. INO80E Is Downregulated in Ox-LDL-Treated Endothelial Cells with Increased Apoptosis

**Figure 2:**
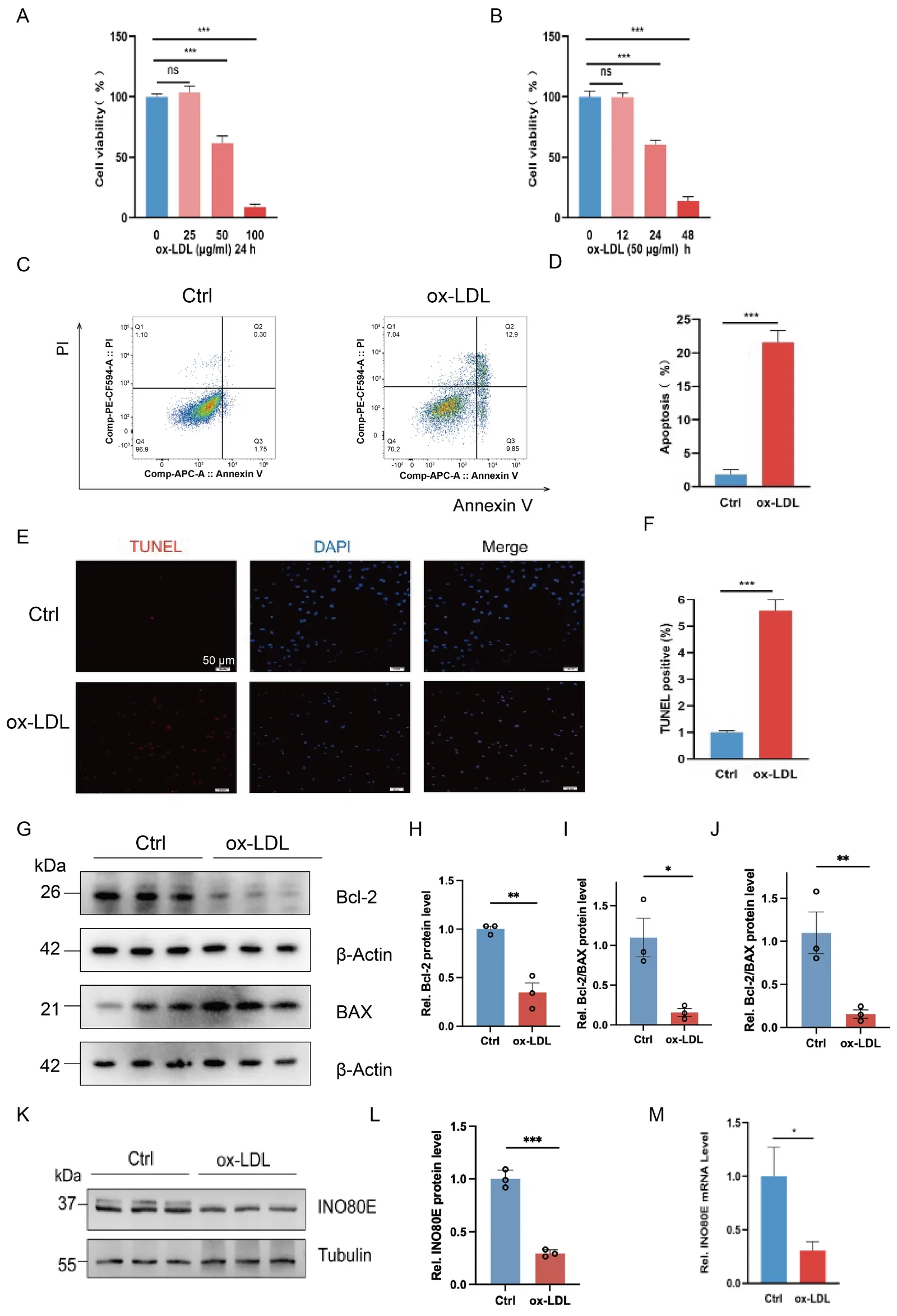
ox-LDL induces endothelial injury and lowers INO80E in HUVECs. (**A**,**B**) CCK-8 responses to ox-LDL concentration and exposure time. (**C**–**F**) Apoptosis measured by Annexin V-FITC/PI and TUNEL. Scale bar, 50 µm. (**G**–**J**) Bcl-2, BAX, and Bcl-2/BAX ratio by Western blotting. (**K**–**M**) INO80E protein and mRNA after ox-LDL. *n* = 3. * *p* < 0.05, ** *p* < 0.01, *** *p* < 0.001.

We then established an endothelial injury condition in HUVECs using ox-LDL. The CCK-8 curves showed dose- and time-associated loss of cell viability, and 50 µg/mL ox-LDL for 24 h produced a reproducible injury window without causing near-complete loss of activity; this condition was therefore used in later functional experiments (Figure 2A,B). This treatment significantly enhanced endothelial apoptosis, as indicated by increased Annexin V positivity in flow cytometry (Figure 2C,D) and increased TUNEL-positive nuclear staining (Figure 2E,F). In parallel, ox-LDL lowered Bcl-2 levels, elevated BAX levels, and consequently reduced the Bcl-2/BAX ratio (Figure 2G–J). Consistent with the in vivo findings, INO80E protein level was significantly reduced in ox-LDL-treated HUVECs, as shown by Western blotting (Figure 2K,L), and this decrease was further confirmed at the mRNA level by qRT-PCR (Figure 2M). Together, these results indicate that ox-LDL-induced endothelial apoptosis is accompanied by downregulation of INO80E in vitro, consistent with the findings in atherosclerotic endothelium in vivo.

### 3.3. Overexpression of INO80E Attenuates Ox-LDL-Induced Endothelial Apoptosis in HUVECs

**Figure 3:**
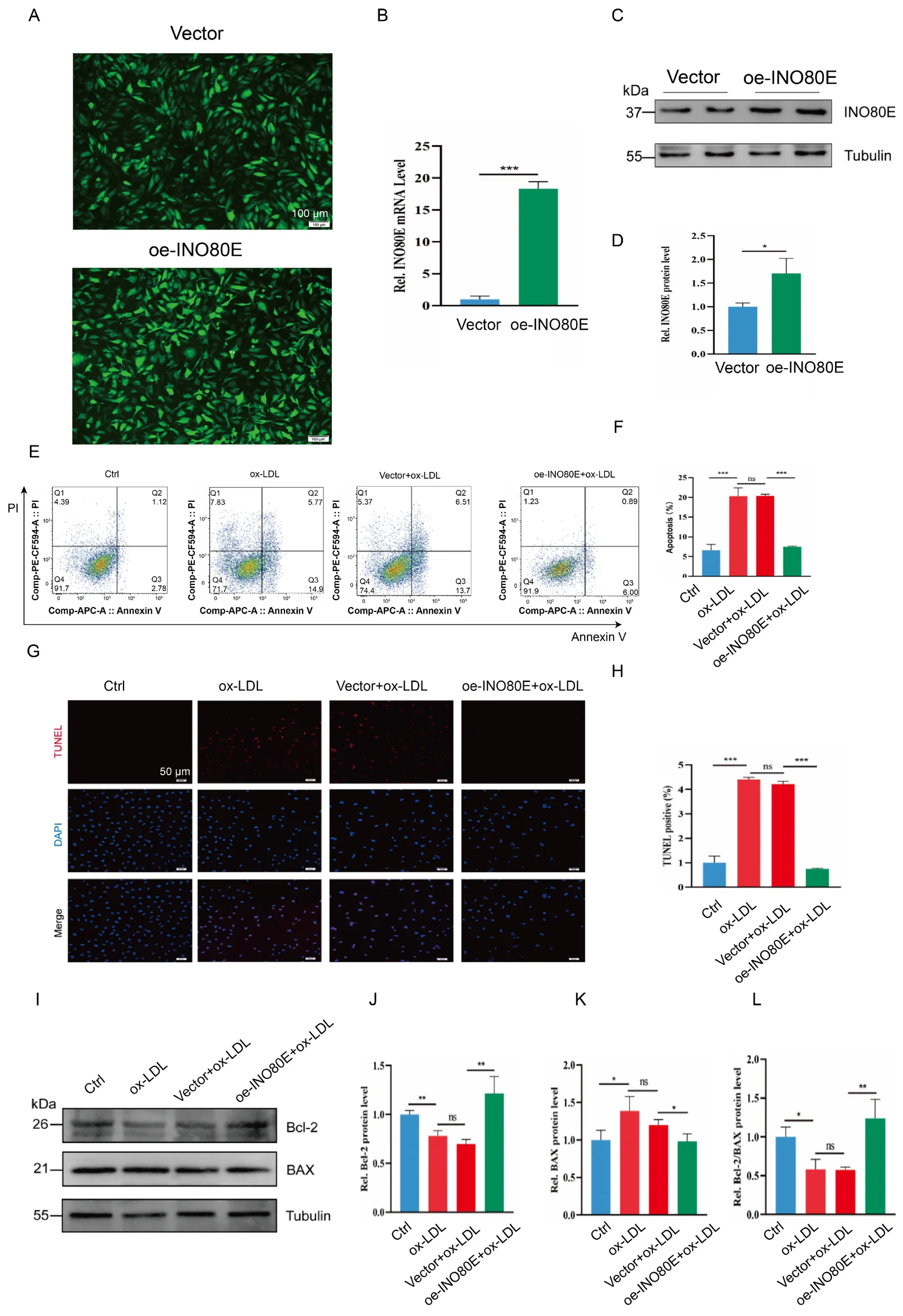
INO80E overexpression attenuates ox-LDL-triggered apoptosis. (**A**–**D**) Efficiency of oe-INO80E transduction assessed by GFP imaging, qRT-PCR, and Western blotting. Scale bar, 100 µm. (**E**–**H**) Flow cytometry and TUNEL analyses. Scale bar, 50 µm. (**I**–**L**) Bcl-2/BAX protein profile. * *p* < 0.05, ** *p* < 0.01, *** *p* < 0.001.

To determine whether reduced INO80E is functionally relevant, we restored INO80E expression in HUVECs before ox-LDL challenge. The lentiviral system produced strong GFP signal and robust increases in INO80E mRNA and protein, confirming successful transduction (Figure 3A–D). When these cells were exposed to ox-LDL, the apoptotic fraction measured by flow cytometry was lower in the oe-INO80E group than in vector controls (Figure 3E,F). TUNEL staining gave the same direction of change, with fewer apoptotic nuclei after INO80E overexpression (Figure 3G,H). At the molecular level, INO80E shifted the apoptotic protein profile by increasing Bcl-2, reducing BAX, and improving the Bcl-2/BAX ratio (Figure 3I–L). Together, these data indicate that INO80E restoration weakens the apoptotic response caused by ox-LDL.

### 3.4. INO80E Interacts with HDAC1 to Facilitate YY1 Deacetylation and Stabilization

**Figure 4:**
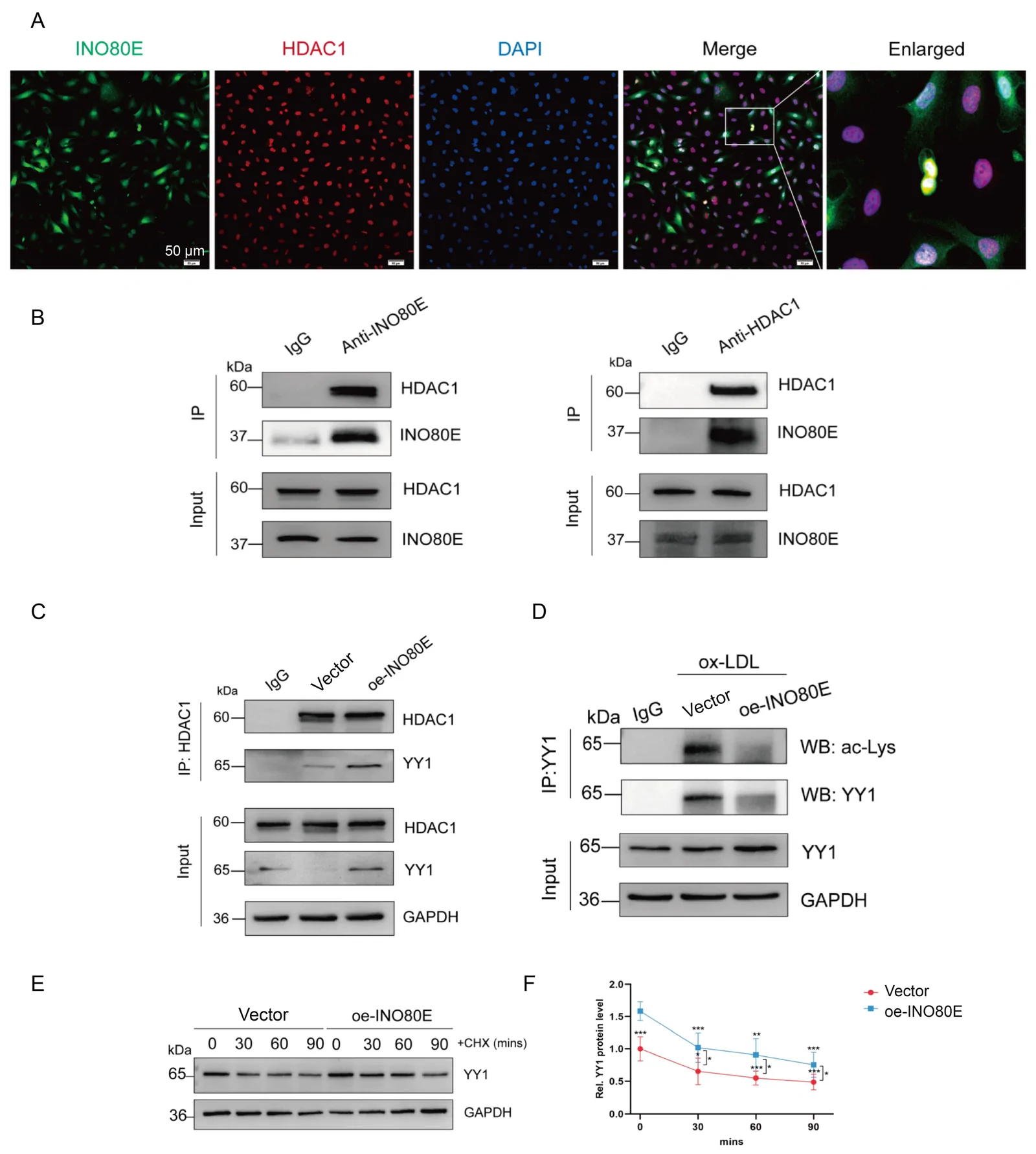
INO80E interacts with HDAC1 and is associated with YY1 deacetylation and stabilization. (**A**) Representative immunofluorescence images of INO80E (green) and HDAC1 (red) in HUVECs. Nuclei are shown by DAPI staining (blue). Merged images indicate their nuclear colocalization. Scale bars: 50 µm. (**B**) Reciprocal co-immunoprecipitation assays showing the interaction between endogenous INO80E and HDAC1. Cell lysates were immunoprecipitated with anti-INO80E or anti-HDAC1 antibodies and immunoblotted with the indicated antibodies. IgG served as a negative control. (**C**) Co-immunoprecipitation analysis of the HDAC1-YY1 interaction in vehicle- or oe-INO80E-transduced HUVECs. Cell lysates were immunoprecipitated with anti-HDAC1, and YY1 was detected by Western blotting. (**D**) Analysis of YY1 acetylation. YY1 was immunoprecipitated from cell lysates and probed with a pan-acetyl-lysine antibody. (**E**,**F**) Cycloheximide (CHX) chase assay for YY1 protein stability. HUVECs were incubated with CHX for the indicated times. Representative Western blots (**E**) and quantification of YY1 protein levels over time (**F**). *n* = 3. * *p* < 0.05, ** *p* < 0.01, *** *p* < 0.001.

To explore the mechanism by which INO80E protects endothelial cells, we examined its interaction with HDAC1. Immunofluorescence staining showed that INO80E and HDAC1 colocalized in the nucleus (Figure 4A). Reciprocal co-immunoprecipitation assays further confirmed their interaction, as INO80E immunoprecipitated HDAC1 and HDAC1 immunoprecipitated INO80E (Figure 4B).

We next examined whether INO80E affects the interaction between HDAC1 and its substrate YY1. Co-immunoprecipitation assays showed that INO80E overexpression enhanced the association between HDAC1 and YY1 (Figure 4C). Consistent with this, YY1 acetylation was reduced in INO80E-overexpressing cells compared with control cells (Figure 4D).

To determine whether reduced acetylation affects YY1 stability, we performed a cycloheximide (CHX) chase assay. INO80E overexpression significantly prolonged the half-life of YY1 protein (Figure 4E,F), suggesting that INO80E stabilizes YY1 through HDAC1-dependent deacetylation. Together, these findings indicate that INO80E enhances the HDAC1-YY1 interaction and promotes YY1 deacetylation and stabilization.

### 3.5. HDAC Inhibition Abolishes the Protective Effects of INO80E in Ox-LDL-Treated HUVECs

**Figure 5:**
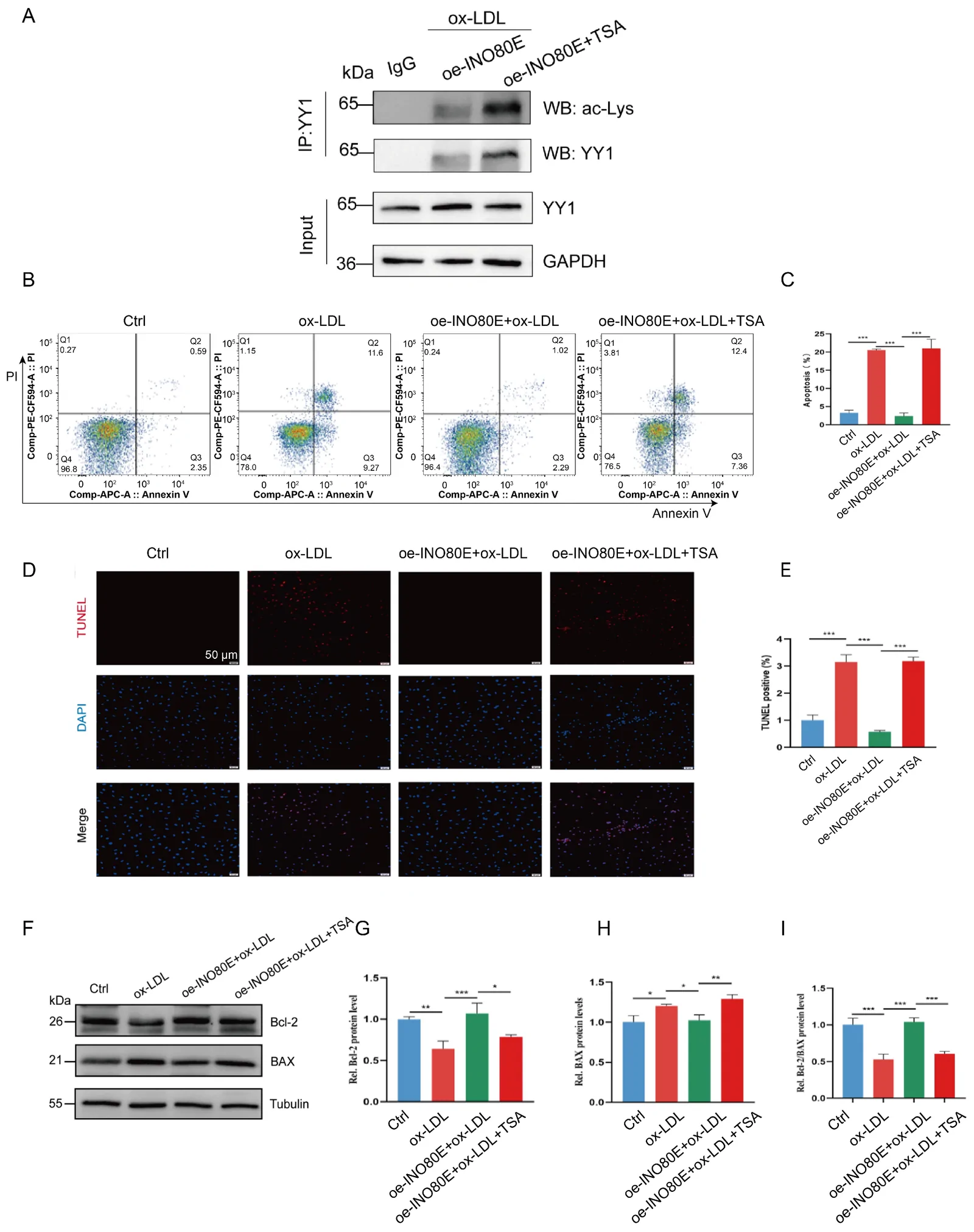
TSA disrupts INO80E-mediated endothelial protection. (**A**) YY1 acetylation after ox-LDL and TSA treatment in oe-INO80E cells. (**B**–**E**) Flow cytometry and TUNEL assessment of apoptosis. Scale bar, 50 µm. (**F**–**I**) Bcl-2, BAX protein profile, and Bcl-2/BAX ratio. *n* = 3. * *p* < 0.05, ** *p* < 0.01, *** *p* < 0.001.

To test whether HDAC activity is required for the INO80E-associated YY1 change, we applied TSA to oe-INO80E-transduced HUVECs. TSA restored YY1 acetylation toward the level observed in vector controls (Figure 5A). This reversal indicates that the decrease in YY1 acetylation produced by INO80E depends on HDAC enzymatic activity rather than on INO80E overexpression alone.

We then examined whether the same HDAC dependence applies to the anti-apoptotic phenotype. In ox-LDL-treated cells, INO80E overexpression reduced apoptosis, but TSA co-treatment largely removed this benefit in both flow cytometry and TUNEL assays (Figure 5B–E). The apoptotic burden in TSA-treated oe-INO80E cells approached that of vector-transduced ox-LDL-treated cells, indicating that HDAC inhibition functionally uncouples INO80E from endothelial protection.

The Western blot results were consistent with the apoptosis assays. TSA prevented the INO80E-associated recovery of Bcl-2, restrained the decrease in BAX, and suppressed restoration of the Bcl-2/BAX ratio (Figure 5F–I). These findings place HDAC activity upstream of the Bcl-2/BAX changes associated with INO80E and support the involvement of HDAC-dependent YY1 regulation in the protective response.

### 3.6. INO80E Knockdown Aggravates the Apoptotic Protein Imbalance, and YY1 Knockdown Attenuates the Protective Effect of INO80E

**Figure 6:**
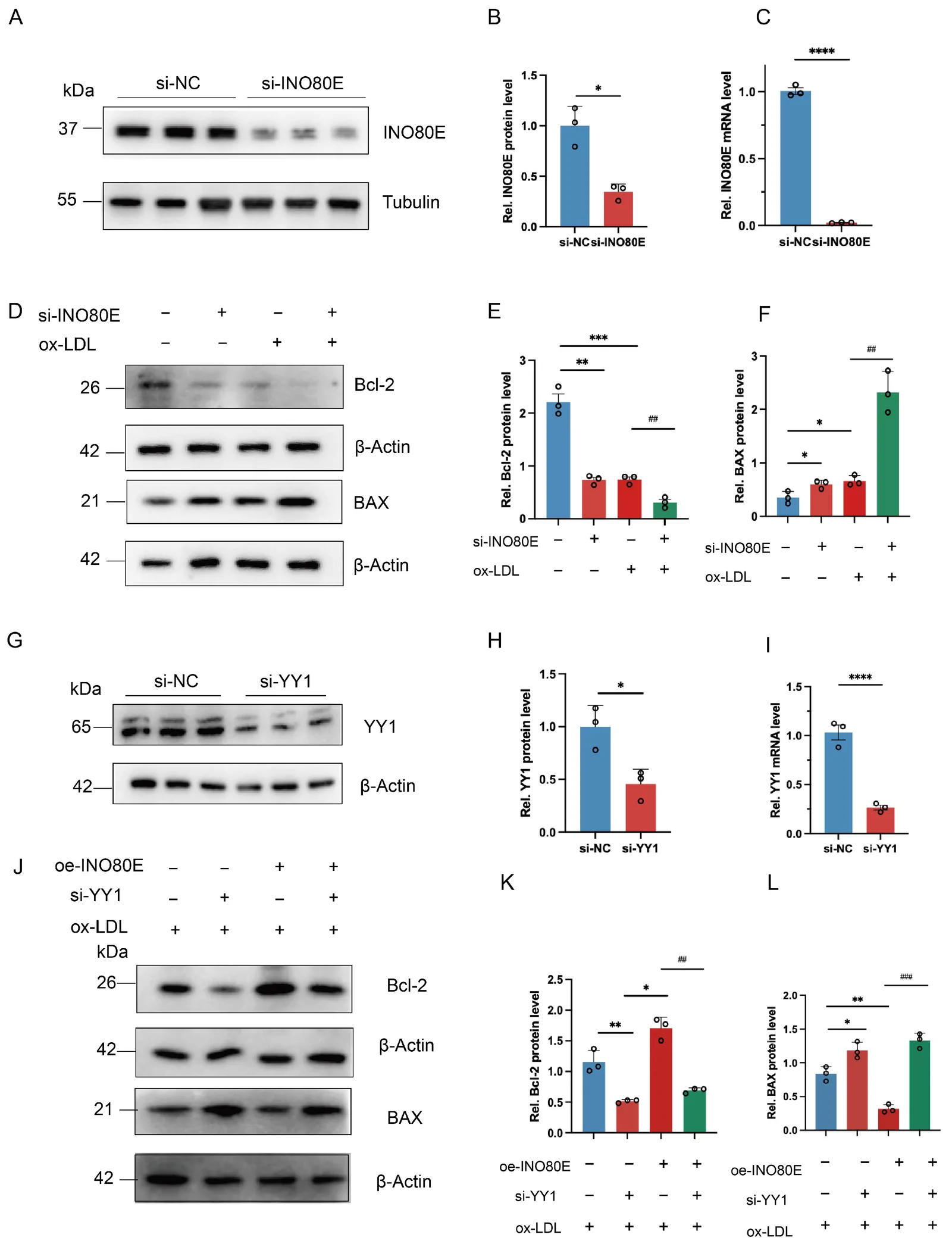
INO80E knockdown regulates apoptosis-related proteins, and YY1 contributes to INO80E-mediated protection. (**A**–**C**) Validation of siRNA-mediated INO80E knockdown in HUVECs by Western blotting (**A**,**B**) and qRT-PCR (**C**). (**D**–**F**) Bcl-2 and BAX protein expression after INO80E knockdown under basal and ox-LDL-stimulated conditions. Representative blots are shown in (**D**), with quantification of Bcl-2 (**E**) and BAX (**F**). (**G**–**I**) Validation of siRNA-mediated YY1 knockdown by Western blotting (**G**,**H**) and qRT-PCR (**I**). (**J**–**L**) Western blot analysis of Bcl-2 and BAX in ox-LDL-treated HUVECs subjected to INO80E overexpression and YY1 knockdown. Representative blots are shown in (**J**), with quantification of Bcl-2 (**K**) and BAX (**L**). *n* = 3. Exact *p* values for selected comparisons were as follows: (**B**), 0.0171; (**C**), <0.0001; (**E**), 0.0061, 0.0008, and 0.0044; (**F**), 0.0354, 0.0238, and 0.0021; (**H**), 0.0229; (**I**), 0.0007; (**K**), 0.0045, 0.0216, and 0.0017; (**L**), 0.0213, 0.0018, and 0.0002. Statistical comparisons are indicated above the bars (* *p* < 0.05, ** *p* < 0.01, *** *p* < 0.001, **** *p* < 0.0001, ## *p* < 0.01, ### *p* < 0.001).

To determine whether endogenous INO80E contributes to apoptosis-related protein regulation, we silenced INO80E in HUVECs using siRNA. Knockdown efficiency was confirmed at both the protein and mRNA levels (Figure 6A–C). Under basal conditions, INO80E knockdown decreased Bcl-2 expression and increased BAX expression. After ox-LDL exposure, these changes became more pronounced, with a further decrease in Bcl-2 and a greater increase in BAX in si-INO80E-transfected cells compared with the corresponding si-NC controls (Figure 6D–F).

We next examined whether YY1 is involved in the protective effect associated with INO80E overexpression. Efficient YY1 knockdown was confirmed by Western blotting and qRT-PCR (Figure 6G–I). In ox-LDL-treated HUVECs, INO80E overexpression decreased BAX expression and restored Bcl-2 expression, whereas concomitant YY1 knockdown partially attenuated these effects (Figure 6J–L). These findings suggest that YY1 contributes to INO80E-mediated regulation of apoptosis-related proteins under ox-LDL stimulation.

## 4. Discussion

Endothelial injury and apoptosis are key contributors to the onset and progression of AS [3,21]. Ox-LDL has been widely used to model atherosclerosis-related endothelial stress, and it can activate several injury pathways, including oxidative stress, ER stress-associated JNK/Mff signaling, and TLR4/NF-kB-driven inflammation [22,23,24]. However, the epigenetic factors that determine endothelial vulnerability to ox-LDL remain incompletely understood.

INO80E is a core component of the INO80 chromatin-remodeling complex, which regulates chromatin accessibility and transcription [11]. Several observations suggest that INO80-related chromatin regulation may be relevant to cardiovascular disease: INO80D loss-of-function variants have been linked to premature AS [25], and genetic analyses have connected INO80E with both neuropsychiatric and cardiometabolic traits [12,26]. These findings provided the rationale for investigating whether INO80E participates in endothelial injury responses.

In this study, we identified INO80E as a potential regulator of endothelial apoptosis under atherosclerosis-related stress. INO80E expression was reduced in the aortic endothelium of atherosclerotic mice and in ox-LDL-treated HUVECs, suggesting that loss of endothelial INO80E may be associated with a pro-apoptotic endothelial phenotype during AS. Functionally, INO80E overexpression attenuated ox-LDL-induced apoptosis, whereas siRNA-mediated INO80E knockdown further reduced Bcl-2 expression and increased BAX expression. These findings indicate that INO80E contributes to the maintenance of an anti-apoptotic protein profile in endothelial cells exposed to ox-LDL.

Mechanistically, our data suggest that the protective effect of INO80E involves the HDAC1-YY1 axis. YY1 has been implicated in endothelial cell survival and Bcl-2 regulation [15,27], and previous studies have reported functional links between INO80-related proteins and YY1 [13,14]. In addition, YY1 stability can be regulated by acetylation-dependent protein degradation [16]. Consistent with these observations, we found that INO80E interacted with HDAC1 and promoted the association between HDAC1 and YY1. INO80E overexpression reduced YY1 acetylation and slowed YY1 protein turnover, suggesting that INO80E may stabilize YY1, at least in part, by facilitating HDAC1-associated YY1 deacetylation.

The YY1 knockdown rescue experiments further support this mechanism. In ox-LDL-treated HUVECs, INO80E overexpression decreased BAX expression and restored Bcl-2 expression, whereas concomitant YY1 knockdown partially weakened these effects. These results suggest that YY1 acts as an important downstream contributor to INO80E-mediated regulation of apoptosis-related proteins. However, the incomplete reversal by YY1 knockdown also indicates that YY1 is unlikely to be the only mediator of INO80E function. Other transcriptional programs, chromatin-associated mechanisms, or stress-response pathways regulated by INO80E may also participate in endothelial apoptosis control.

Several points should be considered when interpreting the proposed mechanism. INO80E is a component of a chromatin-remodeling complex and may therefore regulate broader gene expression programs beyond the HDAC1-YY1 pathway. In addition, TSA is a broad-spectrum inhibitor of class I and II HDACs and may affect the acetylation of numerous histone and non-histone substrates. Thus, although our findings support a role for HDAC-associated YY1 stabilization in INO80E-mediated endothelial protection, they do not exclude additional mechanisms through which INO80E may modulate ox-LDL-induced endothelial apoptosis.

This study has several limitations. HUVECs are fetal venous endothelial cells and may not fully represent adult arterial endothelial cells involved in atherosclerosis [28,29,30,31]. Moreover, donor information for the commercial HUVECs was unavailable, so donor-to-donor variability cannot be excluded. CCK-8 mainly reflects metabolic activity rather than direct cell death [32], and was therefore interpreted together with Annexin V/PI flow cytometry and TUNEL staining. In vivo endothelial INO80E changes were mainly assessed by immunofluorescence, and further validation using endothelial-enriched aortic samples or single-cell approaches would strengthen these findings. Further validation in independent human endothelial donors, adult arterial endothelial cells, matched animal models, and human atherosclerotic specimens will be needed to clarify the relevance of INO80E to atherosclerosis and its potential therapeutic significance.

In summary, our study identifies INO80E as a previously uncharacterized factor associated with endothelial resistance to ox-LDL-induced apoptosis. Reduced INO80E accompanies AS-related endothelial injury, whereas INO80E restoration supports endothelial survival in a manner consistent with HDAC-associated YY1 stabilization. The loss-of-function and rescue experiments strengthen this model but do not establish direct therapeutic relevance. Additional studies using arterial endothelial models, endothelial-specific in vivo systems, plaque outcome measurements, YY1 transcriptional assays, and human atherosclerotic specimens are required before the INO80E-HDAC1-YY1 axis can be considered a validated therapeutic target.

## 5. Conclusions

INO80E protects against ox-LDL-induced endothelial apoptosis through a mechanism consistent with HDAC-dependent YY1 deacetylation and stabilization. Knockdown and rescue experiments further indicate that endogenous INO80E and YY1 contribute to maintenance of the Bcl-2/BAX apoptotic balance. These findings support the INO80E-HDAC1-YY1 axis as a candidate protective pathway in AS that warrants further investigation.

## Data Availability

The original contributions presented in this study are included in the article/Supplementary Materials. Further inquiries can be directed to the corresponding author.

## Supplementary Materials

The following supporting information can be downloaded at: https://www.mdpi.com/article/10.3390/biom16081174/s1, Figure S1. Original immunofluorescence images; Figure S2. Original Western blot images for Figure 2G,K; Figure S3. Original Western blot images for Figure 3C,I; Figure S4. Original Western blot images for Figure 4B–E; Figure S5. Original Western blot images for Figure 5A,F; Figure S6. Original Western blot images for Figure 6A,D,G,J.

## Abbreviations

The following abbreviations are used in this manuscript:

AS	Atherosclerosis
CCK-8	Cell Counting Kit-8
CHX	Cycloheximide
Co-IP	Co-immunoprecipitation
HUVECs	Human Umbilical Vein Endothelial Cells
MOI	Multiplicity of infection
ox-LDL	Oxidized low-density lipoprotein
TSA	Trichostatin A
YY1	Yin Yang 1
