# Supplementary material for: INO80E Suppresses Oxidized LDL-Induced Endothelial Apoptosis Through HDAC1-Mediated Stabilization of YY1
Source: Biomolecules. 2026 Aug 12;16(8):1174. doi: 10.3390/biom16081174 (PMC13510582; doi:10.3390/biom16081174)
Supplement: Supplementary file 1 [file biomolecules-16-01174-s001.zip › biomolecules-4401334-supplementary.pdf]

Figure S1: Original immunofluorescence images.

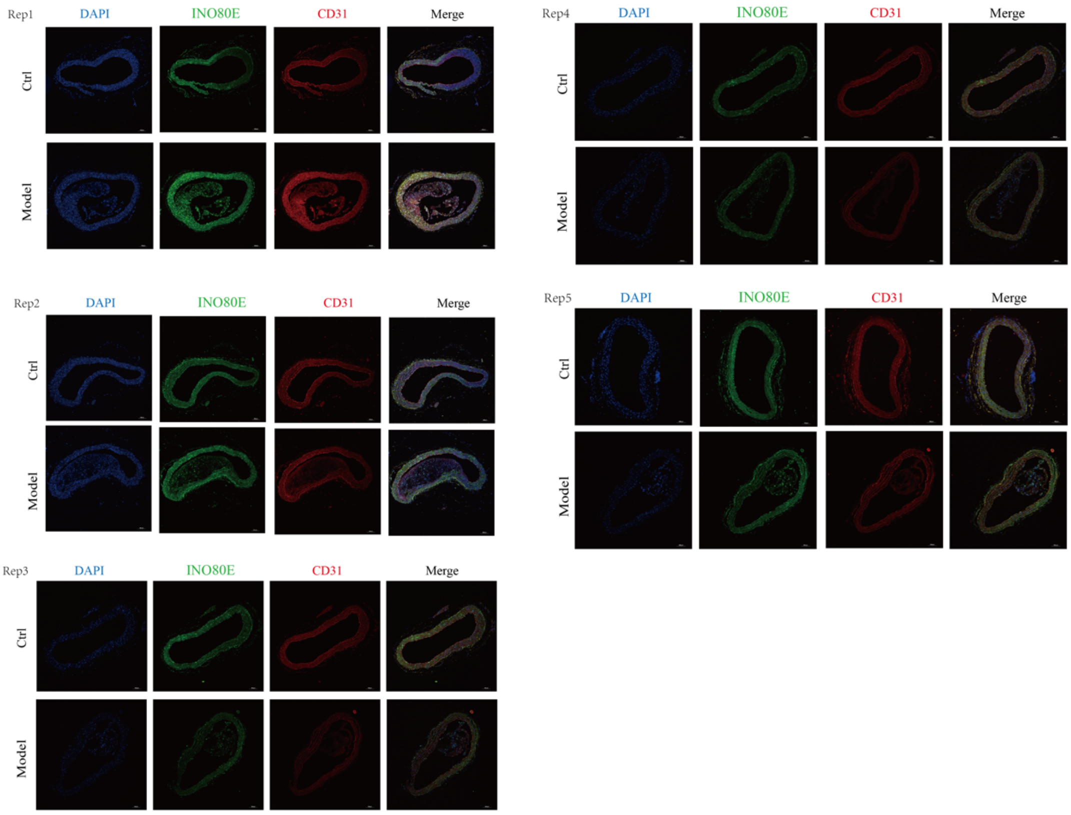

Figure S2. Original western blot images for Figure 2G,K

Figure 2G:

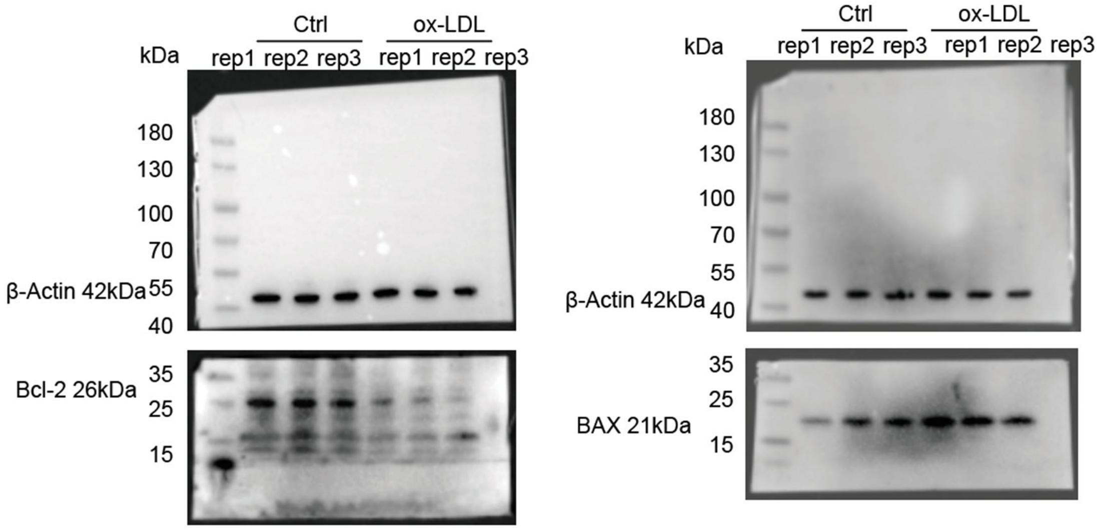

Figure 2K:

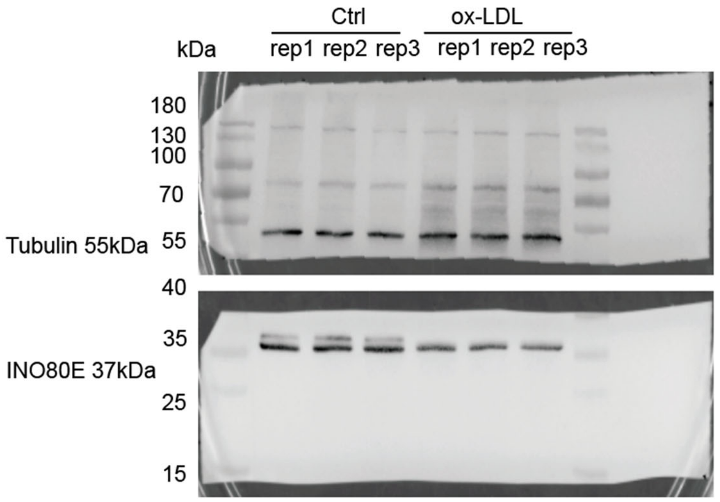

Figure S3. Original western blot images for Figure 3C, I.

Figure 3C:

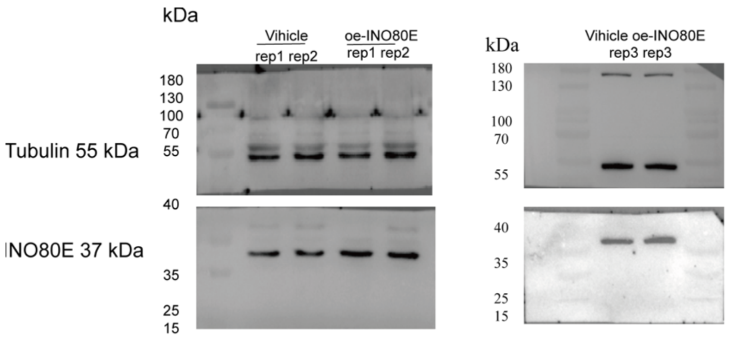

Figure 3I:

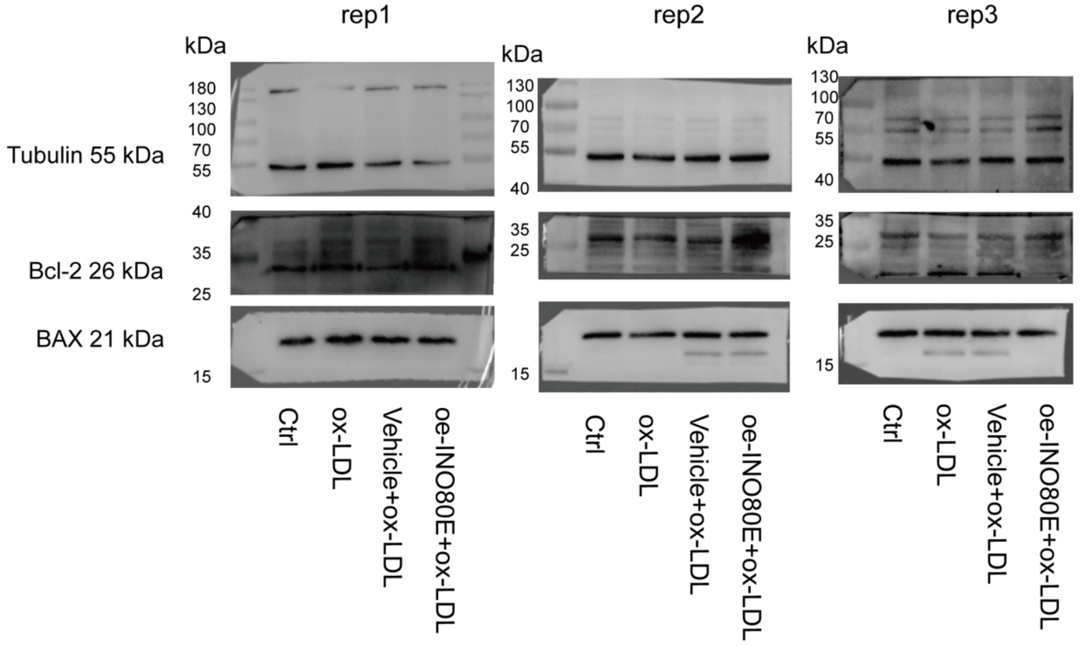

Figure S4. Original western blot images for Figure 4B-E.

Figure 4B:

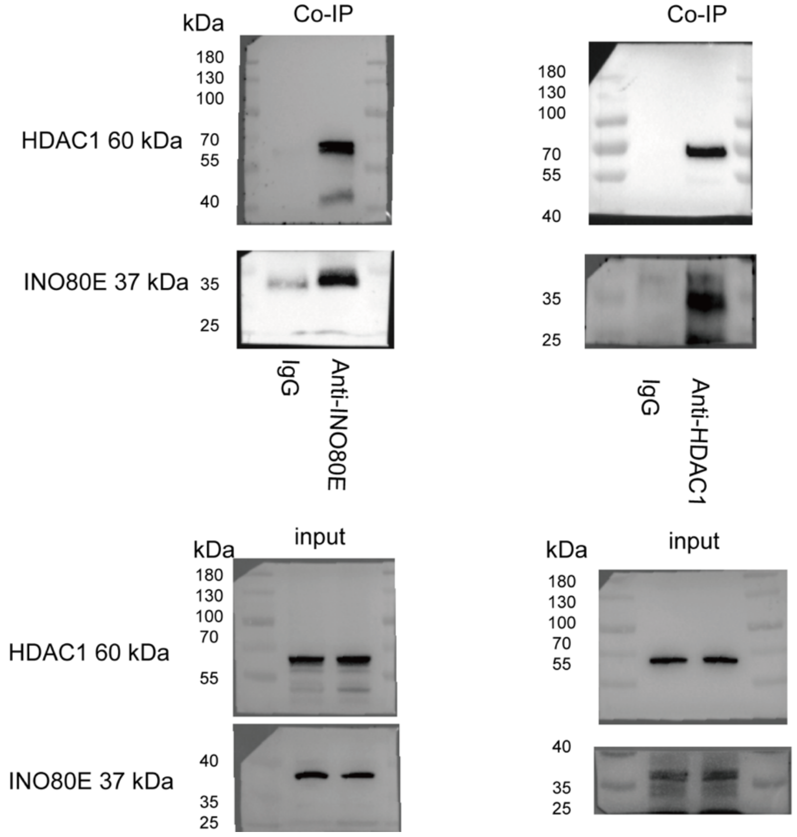

Figure 4C:

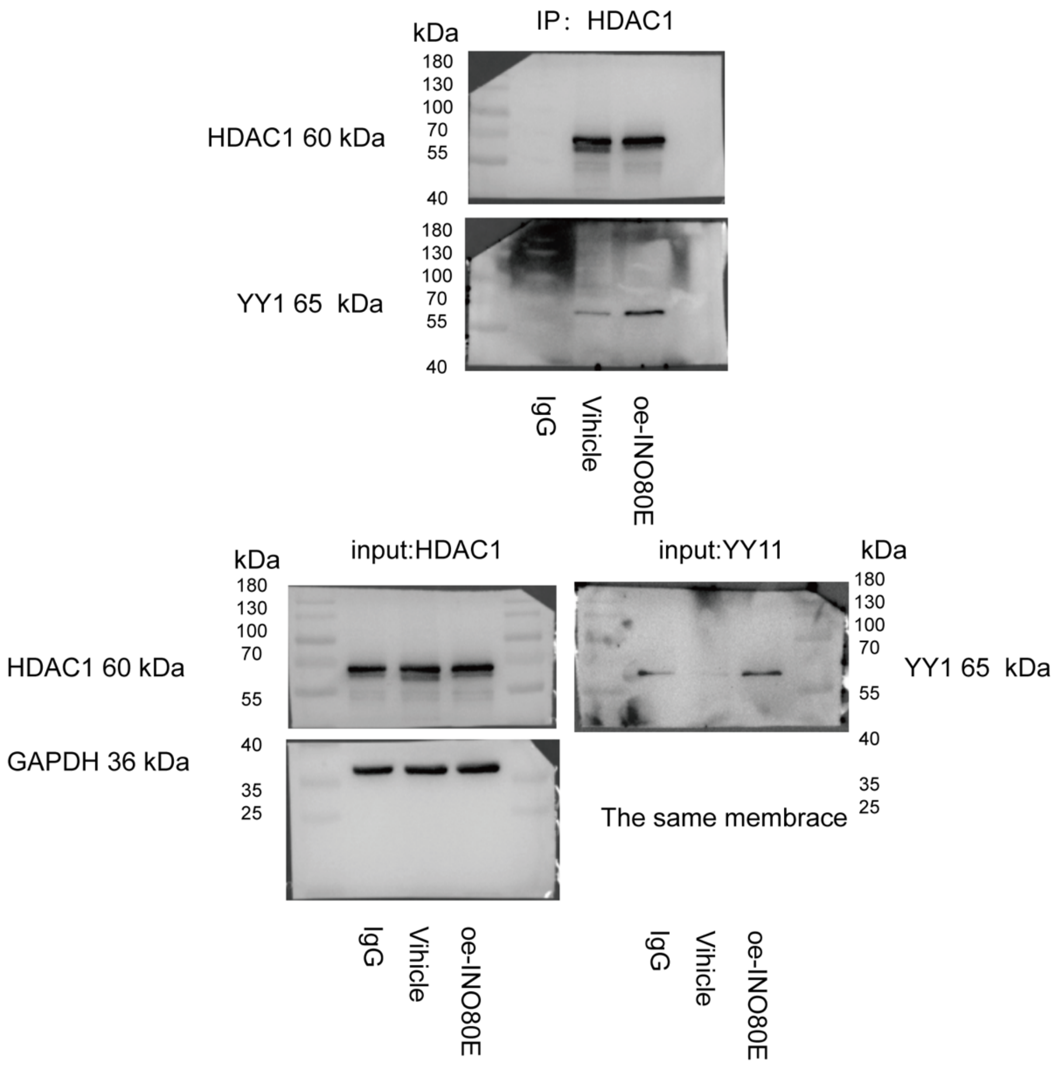
IP：HDAC1
kDa
180
130
100
70
55
40
HDAC1 60 kDa
YY1 65 kDa
IgG
Vihicle
oe-INO80E
input:HDAC1
input:YY11
HDAC1 60 kDa
GAPDH 36 kDa
35
25
YY1 65 kDa
The same membrace

Figure 4D:

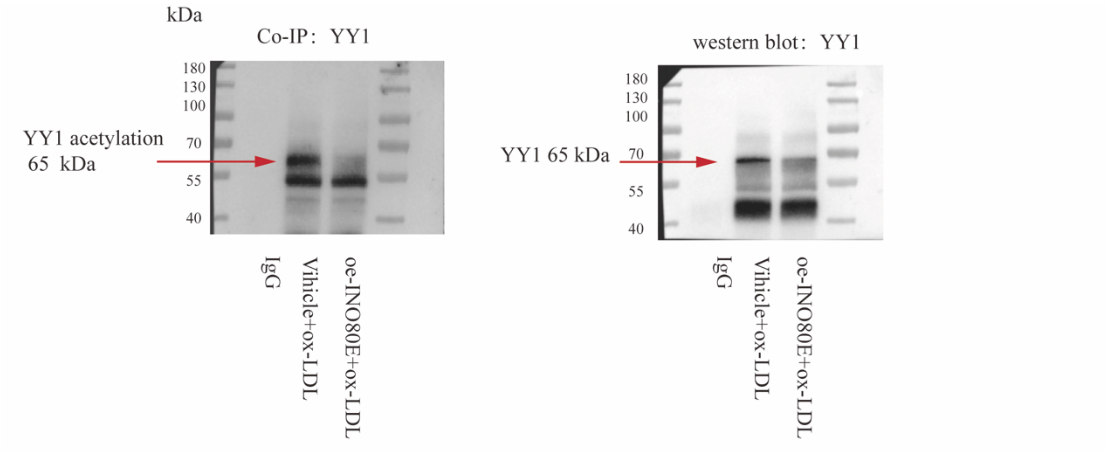
kDa
Co-IP：YY1
180
130
100
70
55
40
YY1 acetylation
65 kDa
IgG
Vihicle+ox-LDL
oe-INO80E+ox-LDL
western blot：YY1
YY1 65 kDa

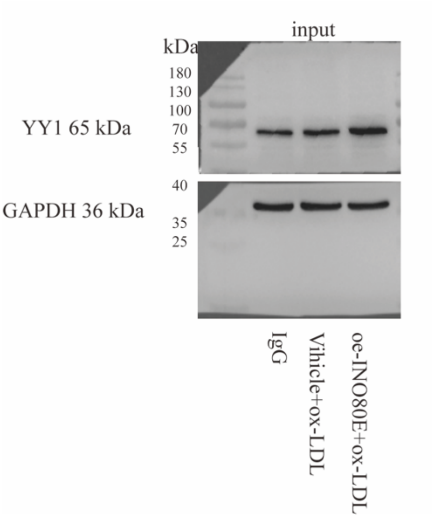

Figure 4E:

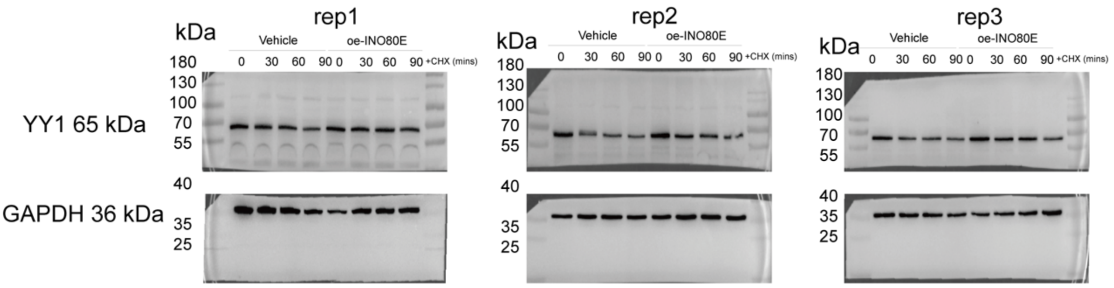

Figure S5. Original western blot images for Figure 5A,F.

Figure 5A:

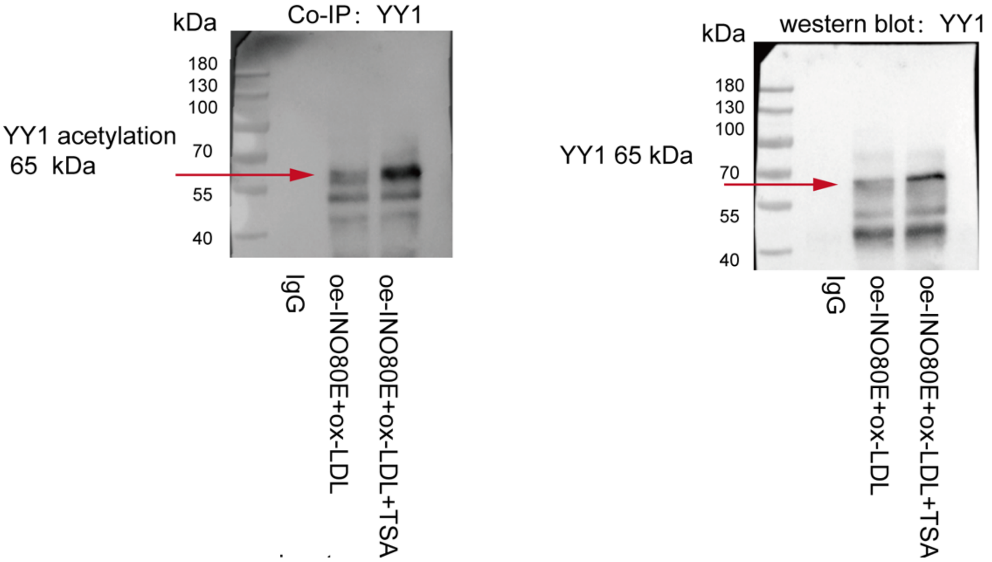

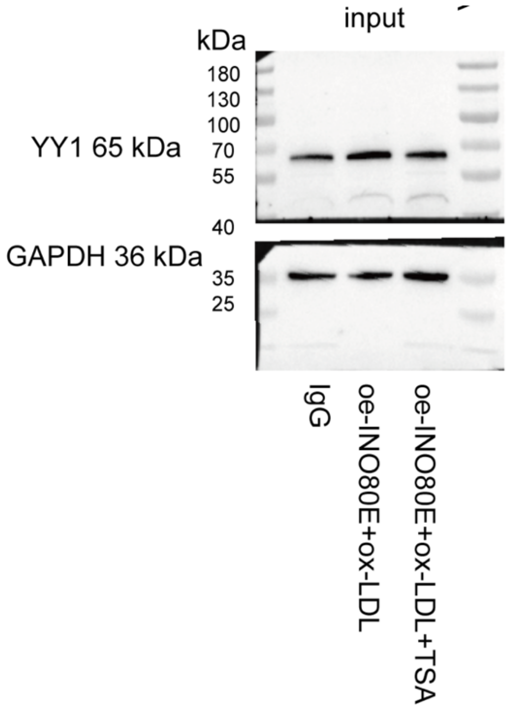

Figure 5F:

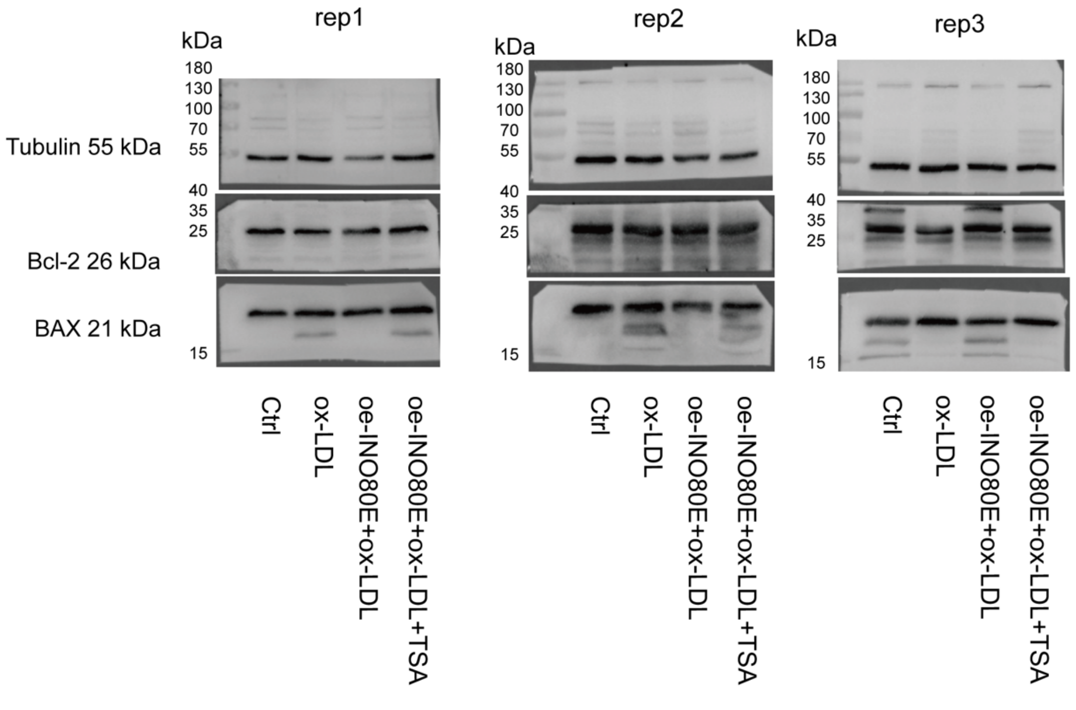

Figure S6. Original western blot images for Figure 6A,D,G,J.

Figure 6A:

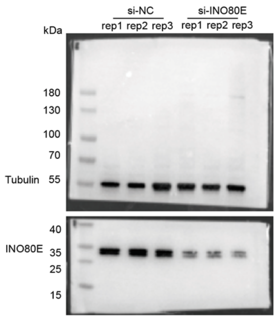

Figure 6D:

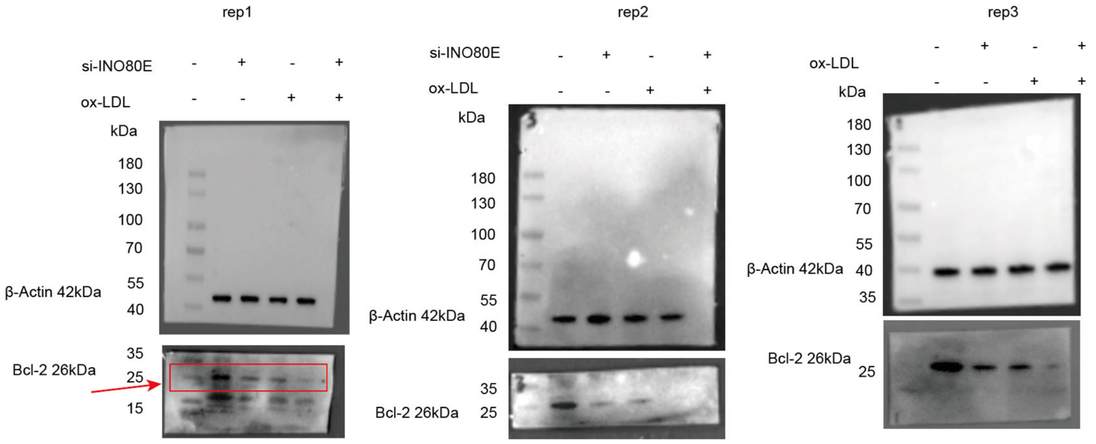

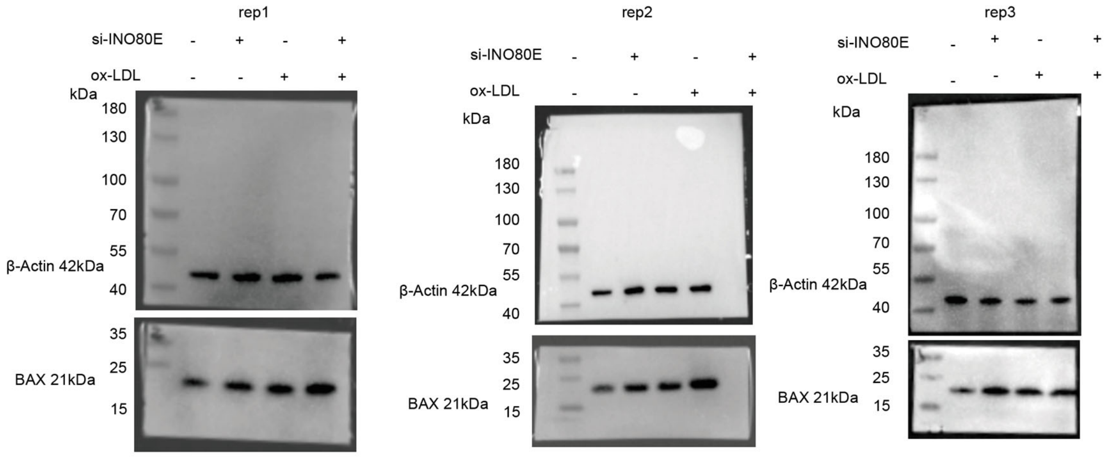

Figure 6G:

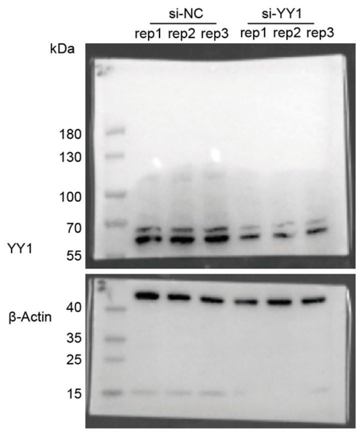
si-NC
si-YY1
rep1 rep2 rep3 rep1 rep2 rep3
kDa
180
130
100
70
YY1
55
40
β-Actin
35
25
15

Figure 6J:

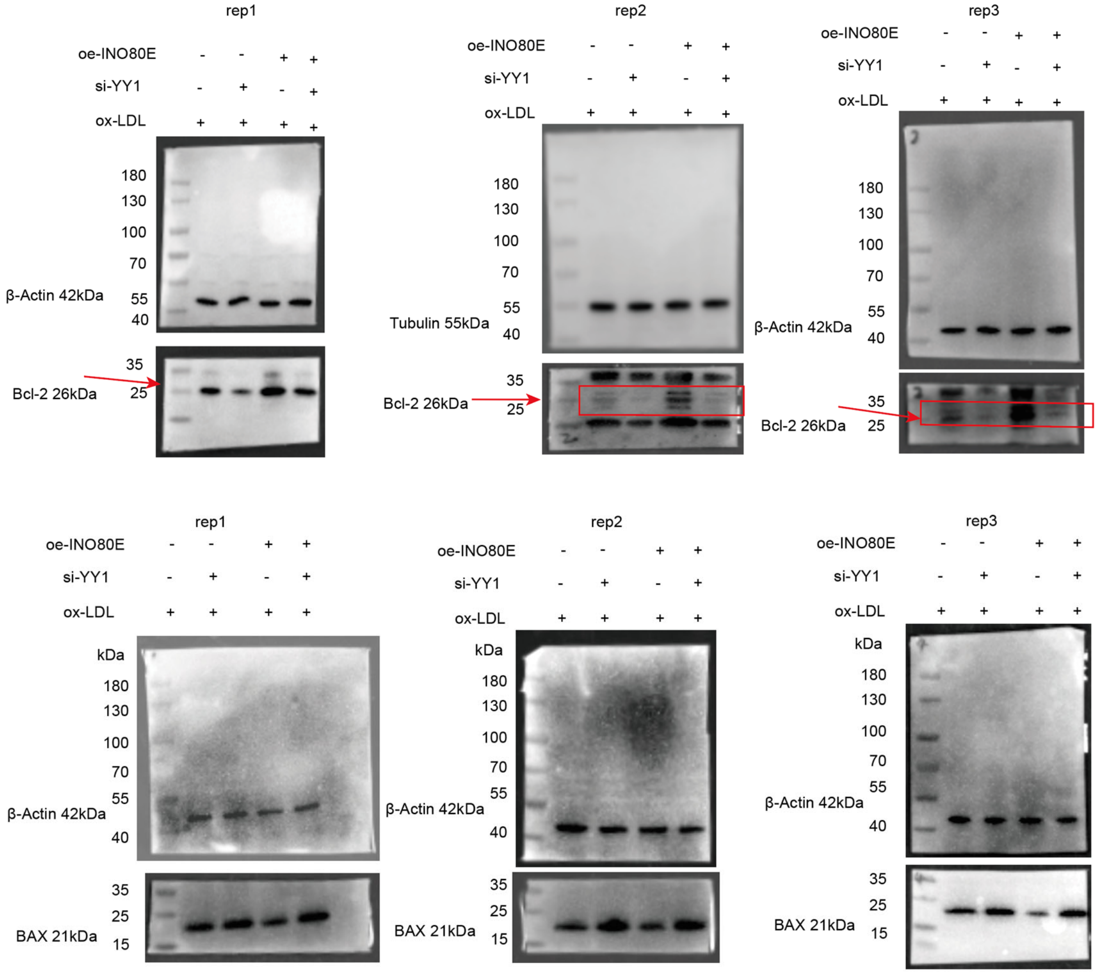
rep1
oe-INO80E - - + +
si-YY1 - + - +
ox-LDL + + + +
180
130
100
70
β-Actin 42kDa
55
40
35
Bcl-2 26kDa
25
rep2
oe-INO80E - - + +
si-YY1 - + - +
ox-LDL + + + +
180
130
100
70
55
Tubulin 55kDa
40
35
Bcl-2 26kDa
25
rep3
oe-INO80E - - + +
si-YY1 - + - +
ox-LDL + + + +
180
130
100
70
55
β-Actin 42kDa
40
35
Bcl-2 26kDa
25
rep1
oe-INO80E - - + +
si-YY1 - + - +
ox-LDL + + + +
kDa
180
130
100
70
55
β-Actin 42kDa
40
35
25
BAX 21kDa
15
rep2
oe-INO80E - - + +
si-YY1 - + - +
ox-LDL + + + +
kDa
180
130
100
70
55
β-Actin 42kDa
40
35
25
BAX 21kDa
15
rep3
oe-INO80E - - + +
si-YY1 - + - +
ox-LDL + + + +
kDa
180
130
100
70
55
β-Actin 42kDa
40
35
25
BAX 21kDa
15
